# Molecular Circuits of Resolution in Renal Disease

**DOI:** 10.1100/tsw.2010.120

**Published:** 2010-07-07

**Authors:** Emma Börgeson, Catherine Godson

**Affiliations:** UCD Diabetes Research Centre, UCD Conway Institute, School of Medicine and Medical Sciences, University College Dublin, Ireland

**Keywords:** lipxoins, resolvins, protectins, renal inflammation

## Abstract

Inflammation is a common feature of renal pathology. Lipid mediators, such as lipoxins, resolvins, and protectins, can actively promote the resolution of inflammation by inhibiting polymorphonuclear cell infiltration to the site of inflammation, shifting the cytokine milieu from proinflammatory to proresolving and increasing the nonphlogistic phagocytosis of apoptotic cells by macrophages. Here we review the evidence for molecular circuits of resolution in renal disease.

## RESOLUTION OF INFLAMMATION

Effective host defense requires an initial inflammatory response that dissipates to ensure tissue homeostasis[1,2]. Conventionally, the dissipation of inflammatory responses was presumed to reflect a decline in proinflammatory mediators. However, over the past decade, there has been a paradigm shift in our understanding of these processes and it is now appreciated that the resolution of inflammation is an actively managed process. A major contribution to our understanding of the resolution of inflammation has been made by investigations of lipid mediator biosynthesis during inflammation[2]. These studies have identified distinct, proresolving bioactions of lipoxins (LXs) and, more recently, resolvins and protectins[3]. Failure of resolution can result in abscess formation, scarring, fibrosis, and eventual organ failure, and subversion of resolution may be reflected in many prevalent chronic diseases, such as arthritis, diabetes, and atherosclerosis[1,2].

An inflammatory insult causes localized production of lipid mediators, such as prostaglandins (PGs) and leukotrienes (LTs), whose proinflammatory bioactions are crucial for host defense. PGs cause vasodilatation, which facilitates the recruitment of leukocytes and monocytes, and LTs are potent chemoattractants that guide the newly recruited polymorphonuclear cells (PMNs) to the site of injury via diapedesis. In this manner, a functional acute inflammatory response arises. Interestingly, it has been shown that the same mechanisms that induce inflammation also program its resolution[4]. The signaling pathways inducing PGE2 and PGD2 formation actively switch the production of lipid mediators from proinflammatory to proresolving by inducing 15-lipoxygenase (LO), which is required for production of LXs, protectins, and resolvins[5]. These proresolving mediators reduce vascular permeability, inhibit PMN recruitment, promote infiltration of monocytes, and stimulate the nonphlogistic phagocytosis of apoptotic

PMNs by macrophages (Mϕ), which is a crucial process in resolution[2] (Fig. 1). It has furthermore been proposed that these proresolving mediators might also stimulate the lymphatic drainage of leukocytes[4].

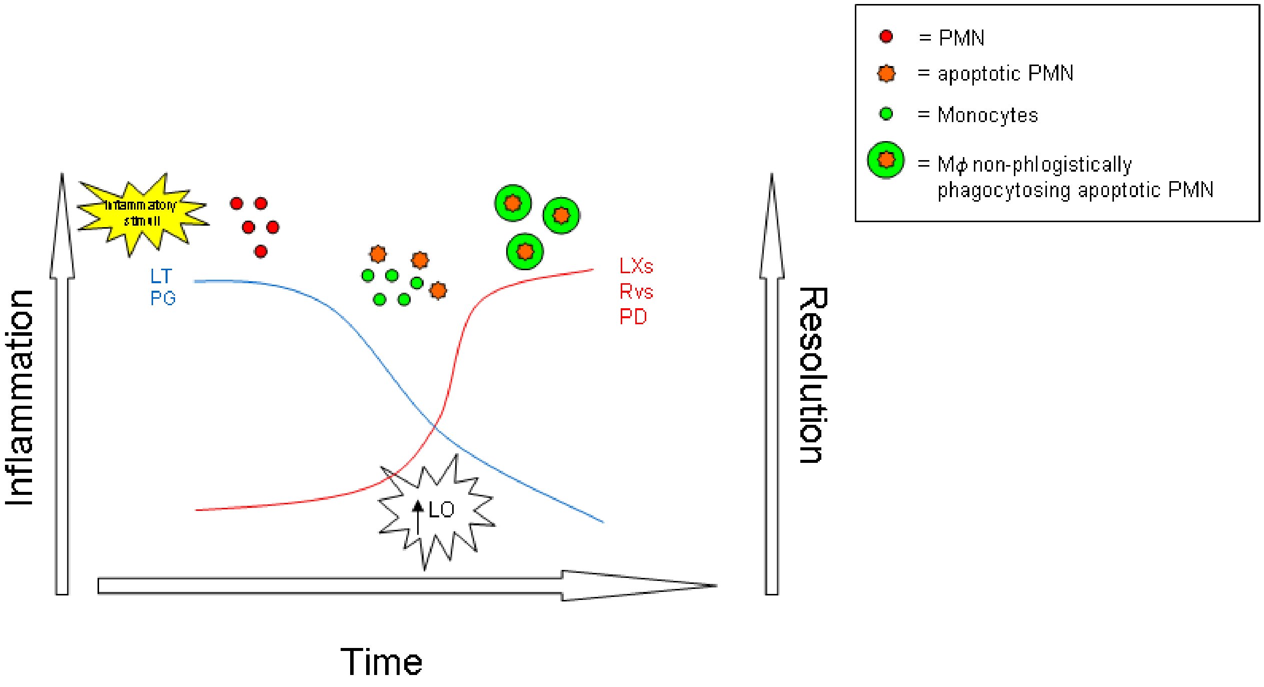

**FIGURE 1.** Temporal lipid inflammation and its resolution. Leukotrienes (LT) and prostaglandins (PG) are synthesized in response to an inflammatory stimulus. These induce vasodilatation and facilitate the recruitment of polymorphonuclear cells (PMNs) into the tissue, which attempt to rid the tissue of the inflammatory stimuli and clear tissue debris. As the inflammation progresses, PGs induce transcription of 15-lipoxygenase (LO) in neutrophils, which induces the formation of lipoxins (LXs), and potentially also resolvins and protectins[5]. These mediators promote the resolution of inflammation by inhibiting PMN infiltration, while stimulating the recruitment of monocytes, inducing a shift in the cytokine milieu and stimulating macrophage (Mϕ) phagocytosis of apoptotic PMN in a nonphlogistic manner[1,2].

Inflammation is a feature of most renal pathology, and severe acute or chronic renal inflammation may lead to glomerulosclerosis, tubular atrophy, damage to renal vasculature, and fibrosis[6]. The pathogenesis of renal inflammation is complex, but irrespective of the initial stimulus, infiltration of neutrophils and monocytes are cardinal signs[6]. Some kidney diseases, such as poststreptoccocal glomerulonephritis, resolve on their own, demonstrating the endogenous ability of repair[7]. In other types of renal disease, such as diabetic nephropathy, the inflammation becomes chronic and causes significant organ injury[8]. Resolution of renal inflammation involves removal of apoptotic cells, leukocytes, and a change of the cytokine milieu from proinflammatory to anti-inflammatory and proresolving[6]. However, the mechanisms by which this occurs and may be subverted in disease are only beginning to be understood. This review aims to clarify some of the mechanisms underlying resolution of renal inflammation, focusing on proresolving lipid mediators such as LXs, resolvins, and protectins.

## LIPOXINS

LXs are endogenously produced eicosanoids with potent anti-inflammatory and proresolving effects. They were discovered in 1984 by Serhan et al. when examining mixed fractions of human leukocytes[9] and were named “lipoxins” since they are **lipox**ygenase **in**teraction product**s**[10]. Lipoxin $A_4$ ($LXA_4$) and its positional isomer lipoxin $B_4$ ($LXB_4$) are the principal species found in mammals.

## Biosynthesis

LXs are generated from the ω–6 fatty acid, arachidonic acid (AA), in a transcellular manner by the sequential action of 5-LO and either 12-LO or 15-LO[11,12] (Fig. 2). They are produced at local sites of inflammation between neutrophils, platelets, and resident tissue cells, such as epithelial cells, where they are active within the pico- to nanomolar range[13,14]. An interesting aspect of LX biosynthesis was discovered in 1990 by Brezinski and Serhan[15] as they learned that leukocytes can be primed for LX synthesis. Their work shows that 15-hydroxyeicosatetraenoic acid (HETE) can be stored in the membrane of neutrophils. Upon activation of the cell, 15-HETE is released and taken up by neighboring leukocytes, which in turn transform it into $LXA_4$ or $LXB_4$[15], indicating that leukocytes may be primed for resolving actions. LX formation can also be induced by aspirin, which, under cytokine-primed conditions, can acetylate cyclooxygenase-2 (COX-2) and thus shift its activity from that of an endoperoxidase to a lipoxygenase[16]. Aspirin inhibits both COX-1 and COX-2 by acetylating a serine residue near the active site, inducing conformational changes that inhibit the oxidation of AA to $PGH_2$, which is the precursor of PGs and thromboxanes. However, although COX-1 is completely inhibited, COX-2 acetylated by aspirin retains catalytic activity to produce 15-HETE from AA, which may in turn be transformed into 15-epi-$LXA_4$ or 15-epi-$LXB_4$ by 5-LO in leukocytes[16]. Interestingly, epi-lipoxins, also called aspirin-triggered lipoxins (ATLs), are even more potent than their precursors and could potentially be responsible for the beneficial effect of aspirin, e.g., in cardiovascular disease[17,18]. Aspirin-stimulated generation of ATLs and inhibition of thromboxane production has been shown to be dose dependent, with low-dose aspirin being most effective[19]. Interestingly, it has been proposed that combined low-dose aspirin, statins, and thiazolidinediones are effective mediators of vessel wall inflammation, whereas high-dose aspirin in combination with these agents has potential adverse effects[20].

## Metabolic Inactivation of Lipoxins

Native LXs undergo rapid inactivation *in vivo,* primarily by PG dehydrogenase–mediated oxidation and reduction[21]. Thus, efforts have been made to design chemically stable LX analogues. The first LX analogues were created in 1995 by Serhan et al.[21] and since then, numerous attempts have been made to design analogues that, while withstanding degradation and inactivation, retain the potent biological activity of native LXs[22,23,24]. Each action of LXs is highly stereoselective[25], and there are three main features that influence the stability and potency of the compound. First, the *R* rather then the *S* configuration at C-15 enhances the activity. This is demonstrated by the fact that the native $LXA_4$ has an *S* configuration, while ATL has an *R* configuration at C-15[26], and the latter molecule is reportedly more potent; for instance, in experimental assays investigating neutrophil migration[27]. It has also been shown that a 5*S*-6*R*-orientation at the two hydroxyl groups, as well as a cis-conformation at C-11, enhances the activity of LXs. One of the first LX-generated analogues made was the 15-epi-16-(*p*-fluoro)-phenoxy-$LXA_4$, which was proven to have many beneficial proresolving effects; for instance, in ischemic acute renal failure[28]. A second generation of LX/ATL analogues was designed where the intent was to avoid metabolism by β-oxidization through insertion of a 3-oxa group. These have a similar biologic activity as the 15-epi analogues, but with a better pharmacokinetic profile[1,29]. More recently, a new type of analogue has been designed featuring a benzo-fused ring system. These have been proven to be as potent as native $LXA_4$ both *in vivo* and *in vitro,* and thus hold potential as therapeutics[30,31].

## Lipoxin Bioactions

LXs have well-established, anti-inflammatory, proresolving, and antifibrotic bioactions, as reviewed in [1,2,32], and have proven to be protective in many models of inflammatory diseases, such as inflammatory bowel diseases[23], periodontal disease[33,34,35], and cardiovascular disease[11]. LXs

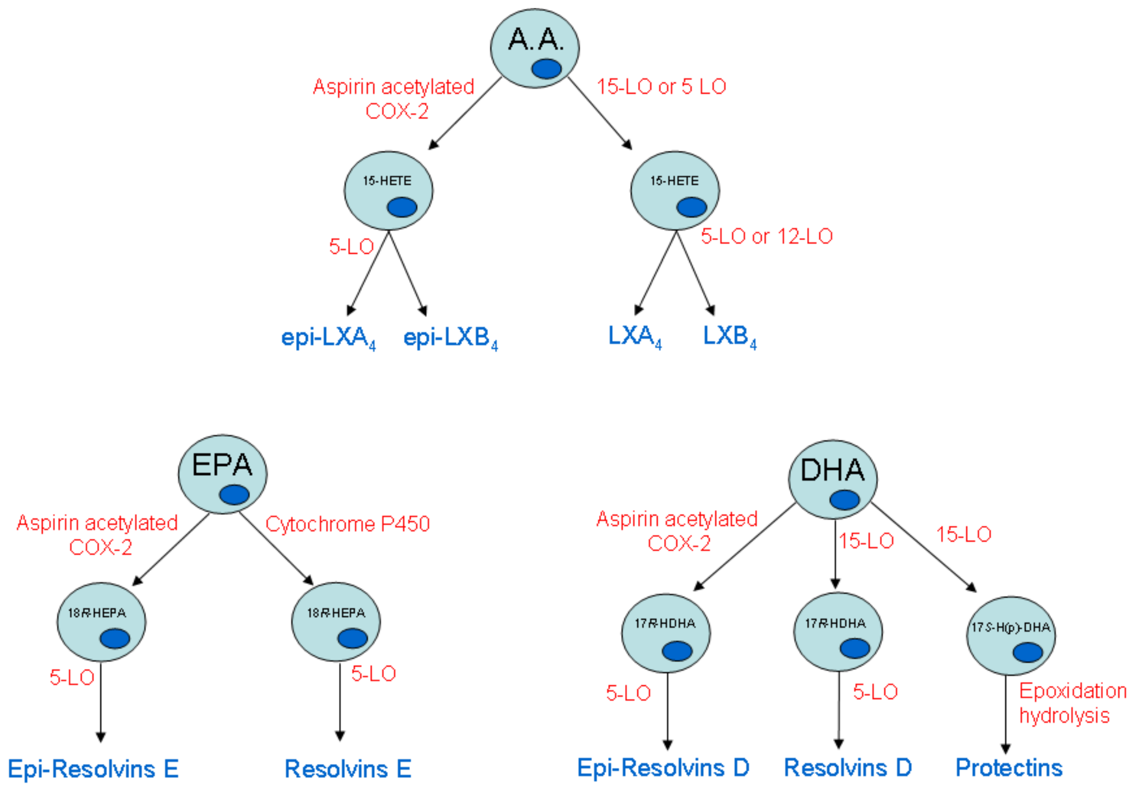

**FIGURE 2.** Biosynthesis of lipoxins, resolvins, and protectins. Lipoxins (LXs) are generated from arachidonic acid (AA) in a transcellular manner involving neutrophils, platelets, and resident tissue cells, such as epithelial cells. AA is transformed by the sequential action of lipoxygenase (LO) into $LXA_4$ or $LXB_4$ with 15-hydroxyeicosatetraneoic acid (HETE) as the intermediate product[11,12]. LX formation can also be induced by aspirin, which acetylates cyclooxygenase-2 (COX-2) and thus shifts its activity from that of an endoperoxidase to a lipoxygenase and enables it to produce 15-HETE from AA, which may in turn be transformed into 15-epi-$LXA_4$ or 15-epi-$LXB_4$ by 5-LO in leukocytes[16]. Resolvins are generated from eicosapentaenoic acid (EPA) or docosahexaenoic acid (DHA). EPA can be enzymatically converted by cytochrome P450 or acetylated COX-2 into 18*R*-HEPA, which can be further transformed by enzymatic epoxidation and 5-LO in leukocytes to form E series resolvins (RvE)[52]. DHA can be converted in a similar manner by the sequential activation of 15-LO or acetylated COX-2 into 17*R*-HDHA, which is then transformed by enzymatic epoxidation and 5-LO to form D series resolvins (RvD)[52]. Protectins are similarly generated from DHA, but via a separate pathway involving 15-LO and enzymatic epoxidation and hydrolysis, where 17*S*-H(p)-DHA serves as the intermediate product[3].

limit leukocyte chemotaxis[36] and inhibit activation of neutrophils and eosinophils[37]. They also stimulate expression of genes involved in resolution[38] and regulate NF-κB activation[39]. Furthermore, they stimulate nonphlogistic Mϕ phagocytosis of apoptotic PMN both *in vitro* and *in vivo*[32,40], which is also associated with a shift from release of proinflammatory to anti-inflammatory cytokines[41]. LXs can also act as vasodilators[42] and can reprogram Mϕ from a classically activated (M1) phenotype to an alternative phenotype[40]. The primary $LXA_4$ receptor first identified in neutrophils by Fiore et al.[43] was a G protein-coupled receptor (GPCR) designated as FPR2/ALX[43,44], which is a receptor that binds pleiotropic ligands, both lipids and small peptides, and elicits either proinflammatory or anti-inflammatory responses[1]. Krishnamoorthy et al. recently found that $LXA_4$ can interact with another GPCR, namely GPR32[45]. It is noteworthy that although $LXA_4$ and $LXB_4$ share biological activities and structures, $LXB_4$ does not bind FPR2/ALX and the $LXB_4$ receptor has not yet been identified. $LXA_4$ has also displayed partial antagonism of a subclass of peptide-LT receptors (CysLTs)[1]. Cross-talk between FPR2/ALX and growth factor receptors also poses a mechanism by which $LXA_4$ indirectly inhibits cellular responses, such as mesangial proliferation, angiogenesis, and fibrosis[1,46,47]. Finally, $LXA_4$ has been suggested to bind the nuclear aryl hydrocarbon receptor (AhR) in dendritic cells, although far greater concentrations of $LXA_4$ are needed for activation of this receptor compared to the GPCR[48]. It is noteworthy that, as many of the bioactions of LXs ascribe an anti-inflammatory and proresolving phenotype, they conversely down-regulate immune responses to pathogens such as *Mycobacterium tuberculosis* and *Toxoplasma gondii*[49,50,51]. These findings emphasize the importance of the temporal and spatial regulations of eicosanoid production in effective host defense.

## RESOLVINS AND PROTECTINS

### Biosynthesis

Resolvins, "resolution phase interaction products", and protectins are generated from the ω-3 polyunsaturated fatty acids (PUFAs) eicosapentaenoic acid (EPA) or docosahexaenoic acid (DHA) (Fig. 2). EPA can, by a series of enzymatic reactions involving cytochrome P450, be converted to 18*R*-HEPA, which can be further transformed by enzymatic epoxidation and 5-LO in leukocytes to form E series resolvins (RvE). DHA can be converted in a similar manner into D series resolvins (RvD) by the sequential activation of 15-LO, enzymatic epoxidation, and 5-LO, where 17*R*-HDHA is the intermediate product[52]. Similarly to resolvins, protectins are generated from DHA, but via a pathway involving 15-LO and enzymatic epoxidation and hydrolysis where 17*S*-H(p)-DHA serves as the intermediate product[3]. As with the ATLs, there are aspirin-triggered epimers of the resolvins where the formation is induced by acetylated COX-2[2].

### Bioactions

Both resolvins and protectins display potent anti-inflammatory and proresolving effects by inhibiting production of anti-inflammatory mediators, regulating neutrophil trafficking, and promoting nonphlogistic Mϕ phagocytosis of apoptotic cells[3,53]. RvE1 was protective in peridontitis, reducing PMN infiltration, preventing bone loss, and promoting tissue healing[54]. They have also been shown to limit neutrophil influx in a murine model of colitis[55], as well as being protective against neovascularization in retinopathy[56]. RvD1 limits PMN infiltration in peritonitis and is protective of experimental ischemia/reperfusion (I/R)–induced kidney injury[57]. Protectin D1 (PD1) has, similar to the other ω-3–derived compounds, proven to be beneficial in peritonitis and kidney I/R injury[57], and also in asthma[29] and ischemic stroke[58]. Furthermore, diminished PD1 has been implicated in neural cell survival in Alzheimer's disease[59]. In neutrophils, RvE1 has been shown to bind BLT1, whereas in Mϕ and dendritic cells, it binds ChemR23[60]. RvD1 has also been reported to interact both with FPR2/ALX and GPR32 in phagocytes[45]. It is, however, as of yet, not entirely clear which receptor the protectins act through.

## THE KIDNEY

The kidneys are unique in receiving high blood flow, approximately 25% of the cardiac output. When the kidneys encounter an insult, adhesion molecules are up-regulated and leukocytes infiltrate the organ and cause inflammation and injury. If kidney injury is not resolved and the inflammation persists, chronic kidney disease (CKD) and end-stage renal failure can develop, with dialysis and transplantation as the only option[61]. In recent years, there has been a massive increase in CKD due to an increase in obesity, hypertension, and diabetes mellitus. Indeed, diabetic nephropathy develops in about 25–40% of diabetic patients and is the major cause of end-stage kidney failure[62]. CKD is characterized by a progressive loss of real function and accumulation of profibrotic extracellular matrix (ECM), which causes glomerulosclerosis and tubulointerstitial fibrosis[63]. Even though CKD is most often diagnosed well before it reaches end-stage kidney failure, there is no treatment as of yet that can halt or reverse the decline in renal function. Since inflammation is a common feature of kidney disease, regardless of initial insult, it may well be worth targeting in drug development, possibly focusing on proresolving mediator such as LXs, resolvins, and protectins.

## LIPOXINS, RESOLVINS, AND PROTECTINS IN RENAL DISEASE

LXs have proven to be proresolving in many types of renal injury, as reviewed in Kieran et al.[64]. LXs play a role in glomerulonephritis, where it reduces proteinuria, glomerular inflammation, and mesangial cell proliferation[65]. Interestingly, both $LXA_4$ and 15-LO are elevated in children with acute poststreptococcal glomerulonephritis throughout the disease process, whereas $LTB_4$ levels peaked during the acute phase and decreased during resolution. Furthermore, $LXA_4$ inhibited $LTB_4$ secretion and also $LTB_4$-induced chemotaxis of PMN isolated from the glomeruli of these children[7]. Leonard et al. have also shown that LXs are also protective in models of acute renal failure (ARF) using a murine I/R model, where an analogue of ATLs gave functional and morphological protection and attenuated chemokine and cytokine responses[28]. Similarly, resolvins and protectins have proven to be important in the resolution of renal disease, attenuating neutrophil influx and Mϕ activation[57,66]. Even though clinical trials are not conclusive, it appears through meta-analysis that treatment with fish oil, which is high in ω-3 PUFAs, reduces the risk of end-stage renal disease in dogs[67] as well as in humans[68,69], and DHA specifically has been shown to be renoprotective in mice[70,71]. Furthermore, An et al. showed, using a rat model of 5/6 nephrectomy, which is a model resembling chronic renal failure, that ω-3 PUFAs reduced up-regulation of proinflammatory and profibrotic pathways and attenuated tubulointerstitial fibrosis[72]. Duffield et al.[57] first showed, using a renal I/R model in BALB/c mice, that endogenous levels of RvDs and PD1 are increased 24 h after I/R injury, both in the kidney and in plasma. Hassan and Gronert[66] confirmed these findings by reporting that dietary ω-3 PUFAs increase endogenous renal expression of PD1 and 17-HDHA, the latter being a metabolic marker for RvDs. Furthermore, when given RvDs and PD1 systemically before I/R, the compounds attenuated ischemic kidney injury in a dose-dependent manner[57]. Interestingly, RvDs were also effective when administered *after* I/R for 72 h, reducing fibrosis at day 15 postinjury[57]. This may suggest that they do not only block proinflammatory events, but also contribute actively to resolution (see Fig. 3).

### Neutrophil Influx

LXs inhibit PMN chemotaxis, adhesion, and migration in numerous pathological conditions, ranging from peridontitis to dermal inflammation, and work as a braking signal for PMN-mediated tissue injury[1]. This has also been demonstrated in the kidney, where LXs repeatedly have been proven to reduce PMN influx and thereby preserve renal function and morphology[28]. Since cellular communication is vital in LX biosynthesis, P-selectin–mediated endothelial-PMN interaction facilitates 15-LO–initiated $LXA_4$ biosynthesis[73]. Interestingly, P-selectin–deficient mice, who have reduced $LXA_4$ biosynthesis by 60%, are more susceptible to glomerulonephritis with a marked increase in PMN infiltration. Furthermore, when attempting to restore $LXA_4$ levels by injecting wild-type platelets, PMN infiltration was reduced and glomerulonephritis less severe[74]. Treating rat PMN with $LXA_4$ *ex vivo* reduces PMN influx to glomeruli in a model of glumerolonephritis[75]. Furthermore, overexpressing 15-LO in rats is protective in experimental glomerulonephritis, reducing inflammation and preserving function, which could be coupled to an increased LX synthesis[76]. Resolvins and protectins also inhibited influx of neutrophils[57], and systemic delivery of PD1 and ATLs reduced PMN recruitment in a mouse model of I/R injury[66].

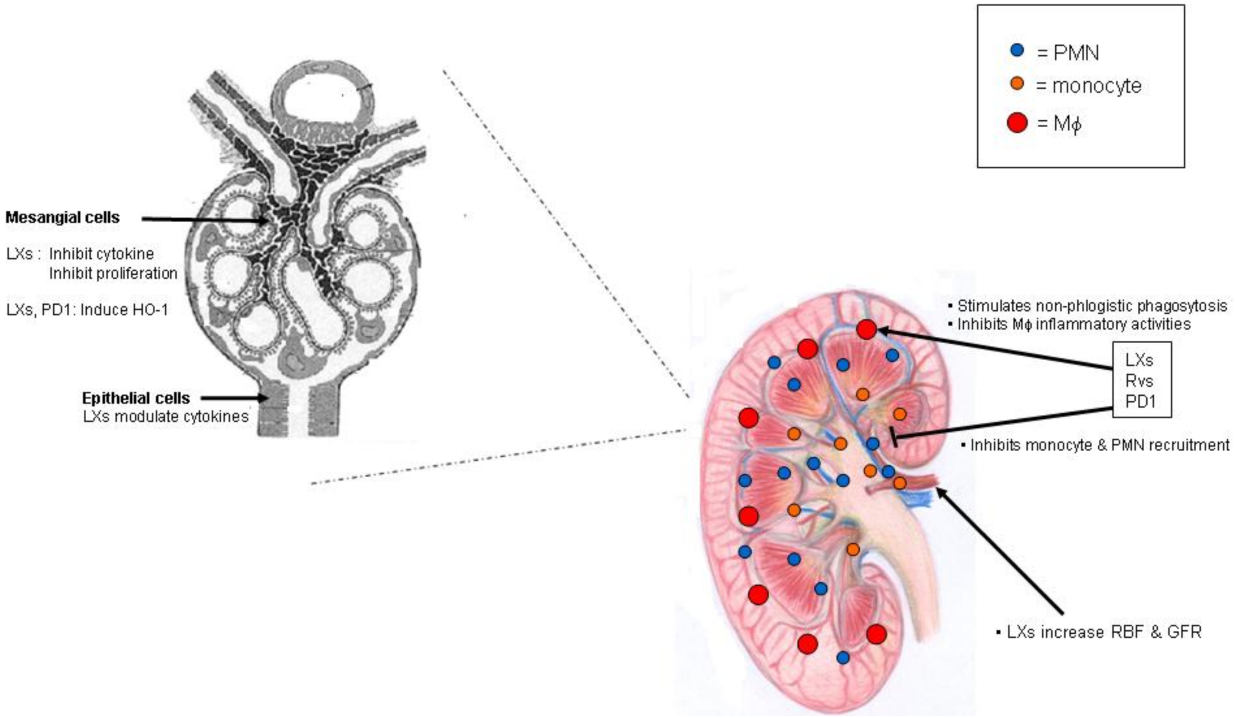

**FIGURE 3.** Bioactions of lipoxins, resolvins, and protectins during resolution of renal inflammation. As renal inflammation proceeds, leukocyte infiltration is pronounced and causes many deleterious effects. Lipoxins (LXs), resolvins (Rvs), and protectins (PD) inhibit the recruitment of polymorphonuclear cells (PMNs) and monocytes[57]. They also stimulate macrophages (Mϕ) to nonphlogistically phagocytose apoptotic cells and inhibit inflammatory activities of Mϕ, e.g., by shifting their cytokine production from inflammatory to resolving[64]. LXs have also been shown to increase renal blood flow (RBF) and glomerular filtration rate (GFR)[107]. Furthermore, LXs modulate cytokine production from both epithelial and mesangial cells, as well as inhibit mesangial proliferation[64]. Finally LXs and PD1 induce the heme-oxygenase-1 (HO-1) pathway, which has been shown to be renoprotective[66]. (Modified from Poussu[133].)

## Macrophages

Most renal diseases are characterized by Mϕ infiltration, which promotes inflammation by secreting mediators such as TNF-α and INF-γ and contribute to the pathogenesis of fibrosis. However, it is becoming increasingly clear that Mϕ also contribute to the resolution of renal inflammation by traits such as clearing apoptotic cells and production of anti-inflammatory mediators such as IL-10[77]. When Mϕ are recruited to a site of inflammation, they develop phenotypically distinctive features in response to local stimuli. Initial descriptions categorize these as either a "classically activated" and inflammatory M1 phenotype, or an "alternatively activated" phenotype[78]. However, recent data suggest that this might be slightly oversimplifying as there are numerous subtypes of the M2 class, and it has been suggested that perhaps Mϕ phenotypes should be grouped into host defense, wound healing, and immune regulating groups instead[79]. Interestingly, Mϕ are not completely committed to their phenotype, but may change or regress to a resting state depending on their microenvironment. For instance an M1 Mϕ can change to a M2 phenotype subsequent to phagocytosis of apoptotic cells[80]. The M2 phenotype has repeatedly been shown to promote resolution[80]. Wang et al. demonstrated the importance of the Mϕ phenotype using a murine model of adriamycin nephropathy, manipulating Mϕ *ex vivo* into either an M1 or M2 phenotype, by stimulating them with either LPS or IL-4/IL-13, respectively. When reinjecting the Mϕ into the mice, they could show that an M1 phenotype induced renal injury, while an M2 phenotype down-regulated inflammatory cytokine and chemokine expression, reduced renal injury, and promoted resolution[81]. Interestingly, LXs have been shown to reprogram cytokine-primed Mϕ with a "classically activated" M1 phenotype to an "alternatively activated" M2 phenotype[40] and could therefore be therapeutically interesting in the context of reprogramming the phenotype of renal macrophages. As previously mentioned, LXs promote nonphlogistic phagocytosis, i.e., the phagocytic process does not provoke release of proinflammatory mediators of apoptotic cells, including PMN and lymphocytes[41]. LXs appear to promote this type of phagocytosis by modulating the actin cytoskeleton, through dephosphorylation of myosin II A and redistribution of Cdc42[32,82]. Defective clearance of apoptotic cells by Mϕ has been implicated in the pathogenesis of chronic nonresolving inflammatory conditions, such as glomerulonephritis[83]. The effect of LXs on the clearance of apoptotic cells in kidney disease needs to be investigated further, but other *in vivo* models have demonstrated the importance of the agent. For instance, LXs promote Mϕ phagocytosis of apoptotic neutrophils in an experimental model of peritonitis[40]. RvDs and PD1 also modulate macrophage recruitment and activity, inhibiting LPS-induced TNF-α production in bone marrow–derived Mϕ *in vitro* and furthermore reducing Mϕ infiltration in an *in vivo* model of I/R[57].

## Dendritic Cells

In the kidney, dendritic cells (DCs) can either be resident or reflect monocyte infiltration. However, their role in renal disease is not entirely understood. In response to I/R injury, DCs increase production of inflammatory cytokines, such as TNF-α, IL-6, MCP-1, and RANTES[84]. In fact, DCs have been identified as the predominant source of TNF-α in the early phase of I/R injury[84]. As antigen-presenting cells, DCs are an important link between innate and adaptive immunity. After first encountering an antigen deemed as pathogenic, they mature and migrate to lymph nodes, where they activate the adaptive immune response. Macconi et al.[85] presented interesting data suggesting that during renal injury, tubular epithelial cells can phagocytose and degrade antigens from the tubular lumen and present them to DCs. These, in turn, mature and migrate to draining lymph nodes, activating CD8+ T cells, which return to the kidney exacerbating tubular epithelial cell injury[85]. Whether or not DCs are influenced by $LXA_4$ is slightly controversial. There is research suggesting that the LX receptor FPR2/ALX is down-regulated as monocytes differentiate into DCs[86]. However, Machado et al.[48] show that $LXA_4$ induces FPRL1 expression in DCs and furthermore triggers SOCS-2 expression. It has also been shown that LXs control DC migration and inhibit excessive production of IL-12[86]. Also, RvE1 has been shown to modulate murine DC IL-12 production[87] and LXs have been shown to negatively regulate LPS-induced differentiation of murine monocytes into DCs *in vitro*[88]. However, how proresolving mediators, such as LXs, resolvins, and protectins, affect renal DCs remains to be established.

## CYTOKINE AND CHEMOKINE EXPRESSION

In renal inflammation and disease, infiltrating Mϕ and lymphocytes are the main source of proinflammatory and profibrotic cytokines[89]. However, resident cells, such as endothelial and mesangial cells, contribute to the production of cytokines and chemokines[90]. LXs are well known to regulate cytokine and chemokine production, and could therefore be important tools in regulating inflammatory mediators in the kidney. Kieran et al. showed in 2003 that ATLs inhibit the expression of many pathogenic cytokines, growth factors, adhesion molecules, and proteases up-regulated in response to renal I/R[91]. For instance, $LXA_4$ inhibits TNF-α–induced production of interleukins[92] and connective tissue growth factor (CTGF) induced MCP-1 and RANTES[47] in rat mesangial cells. In neutrophils, LXs inhibit TNF-α–induced IL-1β, IL-4, and MCP-1[93], as well as LPS-induced IL-8 secretion[94]. As for Mϕ, LXs have been associated with increased TGF-β production, whereas IL-8 is inhibited[41].

NF-κB is a transcription factor vital in the onset of inflammation, regulating the transcription of numerous proinflammatory cytokines, chemokines, and adhesion molecules[95]. However, NF-κB seems to have a dual role in renal pathology, being important both for the inflammatory phase, but also for the resolution. Lawrence et al. first reported that NF-κB appears to promote resolution of inflammation and, using an *in vivo* model of pleural inflammation, showed that whereas blocking NF-κB in the onset of inflammation reduced the inflammatory response, blocking it in the resolving phase had adverse side effects[96]. Panzer et al. later showed more specifically that in renal inflammation, NF-κB has a biphasic activation where the first peak regulates inflammation and the second peak promotes resolution[97]. The first peak is dependent primarily on NF-κB p65/p50 heterodimers, which induce transcription of proinflammatory mediators such as MCP-1 and RANTES. The second peak is regulated by NF-κB p50/p50 heterodimers and seem to down-regulate MCP-1/CCL1 and RANTES/CCL5, as well as TNF-α[97]. In accordance with this, other work has shown that NF-κB p50 can induce expression of anti-inflammatory IL-10 transcription[98], while repressing expression of proinflammatory mediators such as TNF-α[99]. Thus, this body of work suggests that even though it may be beneficial to inhibit NF-κB in the beginning of an inflammatory process, it can be inappropriate to do so during the resolving phase[96]. $LXA_4$ has been shown to inhibit NF-κB in various cell lines. Decker et al. showed that $LXA_4$ inhibits IL-1β–induced degradation of IκBa, which sequesters NF-κB in the cytosol and thus reduces NF-κB activity[39], and has also been found to inhibit NF-κB activation in human synovial fibroblasts[101]. Furthermore, $LXA_4$ analogues inhibit NF-κB p65 activation in human PMN and monocytes[94], and native $LXA_4$ does the same in murine Mϕ[88]. More specifically for the kidney, $LXA_4$ has been shown to inhibit CTGF-induced NF-κB activity in mesangial cells[47] and an $LXA_4$ analogue that was protective in a model of nephritis in rats reduced NF-κB activation[65]. Hudert et al. have shown that ω-3 PUFA reduces NF-κB p50/p65 activity in a mouse model of colitis[102]. Furthermore, PD1 has been shown to inhibit NF-κB p50/p65 expression in a rat model of I/R in the brain[58].

## Mesangial Cells

It is well established that LXs can inhibit PDGF-, CTGF-, $LTD_4$-, and TNF-α–induced proliferation and cell cycle progression in mesangial cells, both *in vivo* and *in vitro*[47,92,103,104,105]. Furthermore, Mitchell et al. showed that $LXA_4$ inhibits PDGF-induced ROS production in human mesangial cells[104], which express the FPR2/ALX[46]. Wu et al. demonstrate the protective effects of LXs in mesangioproliferative nephritis model in rats, where LXs inhibited IL-6 and IL-1β expression as well as reducing α-smooth muscle actin (α-SMA) levels and NF-κB activation[106]. Mesangial cells control renal blood flow and thus also glomerular filtration rate (GFR) via their contractile properties. $LXA_4$ increases renal plasma flow (RPF) and GFR in a dose-dependent manner[107]. Badr et al. also confirmed that $LXA_4$ antagonizes $LTD_4$-induced falls in GFR, however not in RPF[105]. One mechanism by which LXs restore pathological alterations of vascular tone is via inhibiting mesangial proliferation and contractility by antagonizing the cys LT receptor[105] and causing vasodilatation by inducing production of nitric oxide[42].

The heme-oxygenase-1 (HO-1) pathway has proven to be protective in several models of renal disease, both in rodents and humans[108]. The HO-1 enzyme system catalyzes the degradation of toxic heme into bile pigment, biliverdin, and carbon monoxide (CO), which have antioxidant and anti-inflammatory properties and improve blood flow in rat renal transplantation models[109]. HO-1 also regulates gene expression as seen in $HO^{-/-}$ mice, which have increased MCP-1 and NF-κB expression and are more sensitive to I/R injury compared to wild-type animals[110]. LXs increase HO-1 expression in the eye[111] and in endothelial cells[112]. Furthermore, Hassan and Gronert hypothesize that part of the mechanism of action underlying the renoprotective actions of ATLs and PD1 is that they induce the HO-1 pathway, since its expression is increased both *in vivo* after I/R and *in vitro* in rat mesangial cells[66].

## Fibrosis

Renal fibrosis, or tissue scarring, is the final outcome of CKD that does not resolve, regardless of etiology, and pathological features include glomerulosclerosis, tubulointerstitial fibrosis, inflammation, and loss of parenchyma. Fibrosis, in turn, causes end-stage renal failure, which requires dialysis and eventually transplantation. Glomerular mesangial cells, interstitial fibroblasts, and tubular epithelial cells are the major fibrogenic cell types, and infiltrating leukocytes also play an important role[113].

Renal fibrosis is a dynamic process with myofibroblastic activation of mesangial cells and fibroblasts being an early event, and epithelial to mesenchymal transition (EMT) occurring later[113]. Activated fibroblasts, so-called myofibroblasts, are particularly important in renal fibrosis. Recognized by their *de novo* production of α-SMA, they produce excessive amounts of ECM and activate other renal cells to do the same. Renal myofibroblasts can be derived from residing fibroblasts, EMT, endothelial to mesenchymal transition (EndMT), bone marrow–derived cells, or from other mesenchymal cells[114]. Interestingly, $LXA_4$ inhibits IL-1β–induced production of inflammatory cytokines and matrix metalloproteinase (MMP)-3 production in human synovial fibroblasts[100]. Furthermore, recent data from our laboratory indicate that $LXA_4$ can modulate renal fibroblast activation in response to various cytokines, including TGF-β1, as evidenced by abrogated CTGF and α-SMA production in TGF-β1–stimulated renal fibroblasts[115].

Renal fibrosis is characterized by excessive accumulation of ECM, derived from myofibroblasts, mesangial cells, and tubular epithelial cells that have undergone a phenotypic switch and become fibrotic[113]. ECM is primarily made up of components such as collagens, laminins, elastins, and tenacins that often cross-link and become resistant to degradation. It has been hypothesized that the extent of renal fibrosis is due to a balance between collagen synthesis and degradation, where a reduction in the latter results in fibrosis, although new data suggest that this might be slightly oversimplified[114,116]. Most renal ECM degradation is due to MMPs. Interestingly, $LXA_4$ has been shown to prevent IL-1β–induced MMP-3 in human synovial fibroblasts[100] and down-regulates MMP-2 and -9 in human hepatoma cells[117]. Furthermore, an ATL analogue reduced VEGF-induced MMP-9 activity in endothelial cells obtained from human umbilical cord[118].

In renal fibrosis, EMT begins as a part of the repair process after tissue injury and is associated with inflammation, ceasing when inflammation is resolved[119]. However, if the inflammation persists, EMT contributes to fibrosis. During EMT, the epithelial cells of the renal tubules are proposed to acquire a mesenchymal phenotype due to prolonged exposure of inflammatory and profibrotic signals[119]. This increases their migratory abilities and invasiveness, elevates their resistance to apoptosis, and increases production of ECM. The epithelial/mesenchymal cells, together with inflammatory cells and myofibroblasts, induce production of chemokines and MMPs, which cause basement membrane damage and focal degradation of type IV collagen and laminin[119,120]. When the underlying basement membrane is degraded in this manner, the cells can migrate into the interstitium, developing a fibroblastic phenotype and contributing to the pathogenesis of fibrosis, e.g., by producing ECM[121]. Rodgers et al. found that LXs *in vitro* can prevent PDGF-induced EMT in human mesangial cells[122]. Furthermore, LXs have been implicated to play a role in regulating EMT in human pulmonary epithelial cells[123]. Even though RvDs and PD1 seem to primarily affect leukocytes, it is possible that the compounds also effect residing renal cells, although they were unable to protect proximal tubular cells from a swine lineage from oxidative stress[57].

Although TGF-β1 is considered an anti-inflammatory and proresolving cytokine, it is one of the major villains in renal fibrosis and a hallmark of practically all human and experimental forms of CKD[124]. TGF-β1 causes fibrogenic cell activation, ECM accumulation, and EMT[125], and TGF-β1 transgenic mice present with increased apoptosis of podocytes[126]. Furthermore, rats overexpressing TGF-β1 develop glomerulosclerosis and interstitial fibrosis[127], and chronic inhibition of TGF-β1 using TGF-β1–neutralizing antibody inhibits glomerulosclerosis and renal insufficiency in a model of type 2 diabetes[128]. Interestingly, Ma et al. showed, using a nephropathy model, that the effect of TGF-β in the kidney is highly dose dependent as only lower doses of TGF-β–neutralizing antibody improved renal function, seemingly due to decreased glomerular infiltration of Mϕ[129]. However, it should be mentioned that some research suggests that TGF-β may have a much more complex role in the kidney then merely being profibrotic[77]. TGF-β1 seems to deactivate Mϕ to a phenotype that is proresolving, inducing collagen VI, which is a marker of an M2 phenotype[130]. TGF-β1 also induces SMAD-7 signaling, which apart from working as a negative feedback loop on TGF-β signaling, also inhibits NF-κB, thereby reducing renal inflammation and proteinuria[131]. Furthermore, mice overexpressing latent TGF-β1 were actually protective from renal fibrosis in a UUO model, mainly due its anti-inflammatory effects[132]. $LXA_4$-associated phagocytosis is coupled to TGF-β1 release, which is consistent with an immune modulatory phenotype[40]. However, LXs seems to have a different role in renal disease, as Rodgers et al. found that in PDGF-stimulated human mesangial cells, $LXA_4$ reduces TGF-β1 secretion and its downstream targets[122].

## CONCLUSION

Inflammation is a central part of renal pathology and resolution of renal inflammation is vital for the return to normal kidney function following an injury. The resolution of renal inflammation is a complex process, where proresolving lipid mediators, such as LXs, resolvins and protectins, play an important role. LXs, resolvins and protectins actively contribute to the resolution of renal inflammation by inhibiting PMN and monocyte recruitment, shifting the cytokine milieu, and inhibiting mesangial proliferation and possibly EMT. Thus, these proresolving lipid mediators could have great potential as therapeutic targets in renal disease.

## ACKNOWLEDGMENTS

Emma Börgeson is a recipient of an IRCSET Embark postgraduate research scholarship. Work in Professor Godson's laboratory is supported by Science Foundation Ireland, the Health Research Board, and the Government of Ireland Programme for Research in Third Level Institutions.
